# Unraveling the complexities of urban fluvial flood hydraulics through AI

**DOI:** 10.1038/s41598-022-23214-9

**Published:** 2022-11-04

**Authors:** Md Abdullah Al Mehedi, Virginia Smith, Hossein Hosseiny, Xun Jiao

**Affiliations:** 1 Villanova Centre of Resilient Water System, Villanova University, Villanova, PA USA; 2 Department of Earth and Planetary Sciences, Washington University in St. Louis, St. Louis, MO USA; 3 Department of Electrical and Computer Engineering, Villanova University, Villanova, PA USA

**Keywords:** Hydrology, Environmental sciences

## Abstract

As urbanization increases across the globe, urban flooding is an ever-pressing concern. Urban fluvial systems are highly complex, depending on a myriad of interacting variables. Numerous hydraulic models are available for analyzing urban flooding; however, meeting the demand of high spatial extension and finer discretization and solving the physics-based numerical equations are computationally expensive. Computational efforts increase drastically with an increase in model dimension and resolution, preventing current solutions from fully realizing the data revolution. In this research, we demonstrate the effectiveness of artificial intelligence (AI), in particular, machine learning (ML) methods including the emerging deep learning (DL) to quantify urban flooding considering the lower part of Darby Creek, PA, USA. Training datasets comprise multiple geographic and urban hydraulic features (e.g., coordinates, elevation, water depth, flooded locations, discharge, average slope, and the impervious area within the contributing region, downstream distance from stormwater outfalls and dams). ML Classifiers such as logistic regression (LR), decision tree (DT), support vector machine (SVM), and K-nearest neighbors (KNN) are used to identify the flooded locations. A Deep neural network (DNN)-based regression model is used to quantify the water depth. The values of the evaluation matrices indicate satisfactory performance both for the classifiers and DNN model (F-1 scores- 0.975, 0.991, 0.892, and 0.855 for binary classifiers; root mean squared error- 0.027 for DNN regression). In addition, the blocked K-folds Cross Validation (CV) of ML classifiers in detecting flooded locations showed satisfactory performance with the average accuracy of 0.899, which validates the models to generalize to the unseen area. This approach is a significant step towards resolving the complexities of urban fluvial flooding with a large multi-dimensional dataset in a highly computationally efficient manner.

## Introduction

Flooding is globally one of the world’s most destructive types of disasters. In the coming years, floods are expected to be more frequent and larger globally^1–3^. Coupled with the effects of rapid urban growth and climate change, the frequency of large fluvial flooding events is expected to increase, elevating the destructive impact of floods^4–9^. To untangle this challenge, engineers, planners, and emergency managers must be able to accurately anticipate flood extent and depth^10^. Alterations to the depth and time of occurrence of precipitation as a result of climate change are forecasted to reshape the flooding scenarios encountered in many areas in the world shifting flood risk^11^. These risks are also driven in part by local stormwater management and fluvial infrastructure, making predicting flood events a particularly arduous and critical challenge in the built environments^12,13^. With the increase in urbanization, more impervious areas are generated resulting in less infiltration and greater flood peaks and runoff^14^. Hydrological response time is largely reduced in an urban setting increasing fluvial flood risk where the amount of impervious surface area is high^15^. As a result, assessing flood risk in urban areas involves a complex interaction between natural and engineered processes, some of which operate at very local scales, requiring fine-resolution data^16^. Numerous investigations have sought to define the pattern of hydrological regime transformation resulting from urban development^17^. Alteration in urban river flow regimes is ascribed to the construction of impervious areas which facilitate rapid surface runoff from rainfall, the drainage of surface runoff through sewers to the river, and fluvial infrastructure^18–20^. The proportion of urban land cover or the proportion of impervious cover within a catchment area provides predictor of changes in hydrograph characteristics, that lacks precision (e.g., the widely used Curve Number method)^21,22^. Hydraulic models provide more precise results, demand expensive computational results and data.

The complex and ever-changing urban landscape makes urban fluvial flood prediction and modeling computationally expensive and often infeasible due to high-resolution data requirements. Numerical analysis of hydraulic equations across spatio-temporal boundaries can be increasingly expensive depending on the resolution needed. In addition to the complex non-linear relationship among the features of urban fluvial flooding, estimating hydraulic parameters using physics-based equations is computationally expensive, as this process requires a large amount of memory allocation^23^. Due to the computational expense, the range of input data is often limited, resulting in models at course resolutions, exclusive to different types of hydraulic conditions and potentially relevant parameters. Moreover, the uncertainty of the parameters, defective model calibration and errors in the measurements can serve as an accelerator to the computational expense. Answering the challenge of urban fluvial flood necessitates models that can efficiently and effectively represent flood extent with available data, in a quick and robust manner.

Data-driven prediction with Machine Learning (ML) techniques in the field of artificial intelligence (AI) provides a potential solution. ML is rapidly growing in popularity across many fields. ML methods, including the emerging deep learning (DL) methods, have been successfully applied to the field of water resources for stage-discharge (Q/h) relationships^24^, rainfall-runoff^25^, sediment transport^26,27^, flood prediction^28^, water quality analysis^29^. AI models are specifically convenient when the uncertainty in model parameters, complexities in the physics-based equations and computational efforts are significantly high^30^, such as in urban hydrology. Several previous studies have used ML models for urban pluvial flood detection^31–33^. For example, a coupled physic-based model and random forest algorithm has been used to detect flood-prone areas in an urban coastal community^34–37^. The deep convolutional neural network was used to forecast long-term water levels using rainfall intensity with slope and surface curvature^38^. However, a convolutional network takes a long time and requires tedious hyperparameter optimization for the entire stochastic process, particularly when using large datasets^39,40^. Neural network models are highly sensitive to the initial randomization of weights, number of layers, number of neurons, activation functions and algorithm to choose (e.g., gradients descent)^41–43^. In the traditional ML and DL methods, a major challenge lies in developing models that can generalize to unseen case studies and sites^44^. This investigation overcomes this obstacle by leveraging a two-stage approach with a set of ML classifiers and a DNN-based regression model used to predict the flooded extends and magnitudes with a comprehensive set of urban hydraulic features. In several previous studies, data points were randomly divided into a training/testing set for the ML models with satisfactory performance^45–48^. However, due to the spatial autocorrelation effect, random sampling may not be adequate to validate the models to generalize to perform in the unseen area. Therefore, in this study, the models are tested considering the entire study domain as well as cross-validated spatially to minimize the spatial autocorrelation effect using the blocked K-folds Cross-Validation (CV) technique.

To predict the fluvial flooded locations, a set of classifiers e.g., Logistic Regression (LR), Support Vector Machine (SVM), K-nearest Neighbor (KNN) and Decision Tree (DT) are used. Binary classification generates the output in the form of binary data i.e., 0 s and 1 s, which represent whether a location is flooded or not in the study area. LR model has shown satisfactory performance in the previous study in classifying flooded locations using the matrix of the probability of detection on average for flood events^49,50^. The DT algorithm showed good performance with Minimum Absolute Error (MAE) and classification accuracy in IoT (Internet of Things) based flood detection and notification system^51,52^. The SVM attributes the non-linear transformation of geographic and hydraulic features in higher dimensional feature space^53,54^. Highly satisfactory performance was achieved from the SVM algorithm in detecting the area prone to flood risk in the river basin of Buzau in Roman^37^. Supervised regression with Deep Neural Network (DNN) is performed to predict the water depth within the model domain. Real-valued regression with an artificial neural network provides a reliable means of predicting the flooding depth with a good performance range^55–57^. In this investigation, a multilayer perceptron-based feedforward neural network with a back propagation algorithm is used to perform the regression task i.e., predict water depth in the computational space. The DNN regression model is specifically suitable for predicting the flooded depth with representative geographic and hydraulic features^58^. To increase the efficiency of the entire process of flood prediction, an efficient ML workflow plays a vital role by minimizing human involvement and increasing automation through coding^59,60^. Several previous research works predicted flooding using various features e.g., elevation, slope, aspect, curvatures, topographic wetness index, and hourly rainfall^61–64^. However, no investigation was performed to incorporate features that are closely related to the flooding in the urban environment e.g., fluvial infrastructure, impervious location within the contributing area. Many previous studies have shown their importance in runoff calculations. Urban streams and rivers are highly complex and particularly sensitive to urban land use and land cover areas^65–69^, stormwater management^65,70^, and the presence of fluvial infrastructure^71,72^ in addition to the geology and climate of the watershed. To represent the urban environment, we incorporated the effect of the impervious portion within the contributing area in the models. As a part of urban hydraulics, we introduced the downstream distance of the stormwater outfall and dams within the study area. All the variables under the topography, land covers and fluvial infrastructures linked to the urban flood dynamics were chosen based on an extensive literature search to accurately represent the urban hydrologic environment, without double counting variables^73–76^. This study delineates a novel data-driven strategy toward unraveling the complexities of the urban flooding environments using multiple AI approaches i.e., a set of binary classifiers to detect flooded locations and DNN regression to predict water depth with blocked K-folds Cross-Validation (CV). The approach incorporates the characteristics of the urban area by introducing urban hydraulic drivers (impervious locations within the contributing area) in training the models for prediction. The outcomes of this research have significant potential to advance the flood preparedness mechanism for urban areas vulnerable to riverine flash floods where devastation due to rapid accumulation of flood water is significantly high. The quick and flexible framework presented here is transferable and can be utilized to prepare large-scale flood maps in an inexpensive and efficient way in the cloud-computing platform across urban areas. The approach outlined in this study has the potential to efficiently predict urban fluvial flooding for a range of scenarios.

## Data and methods

### Study area

**Figure 1:**
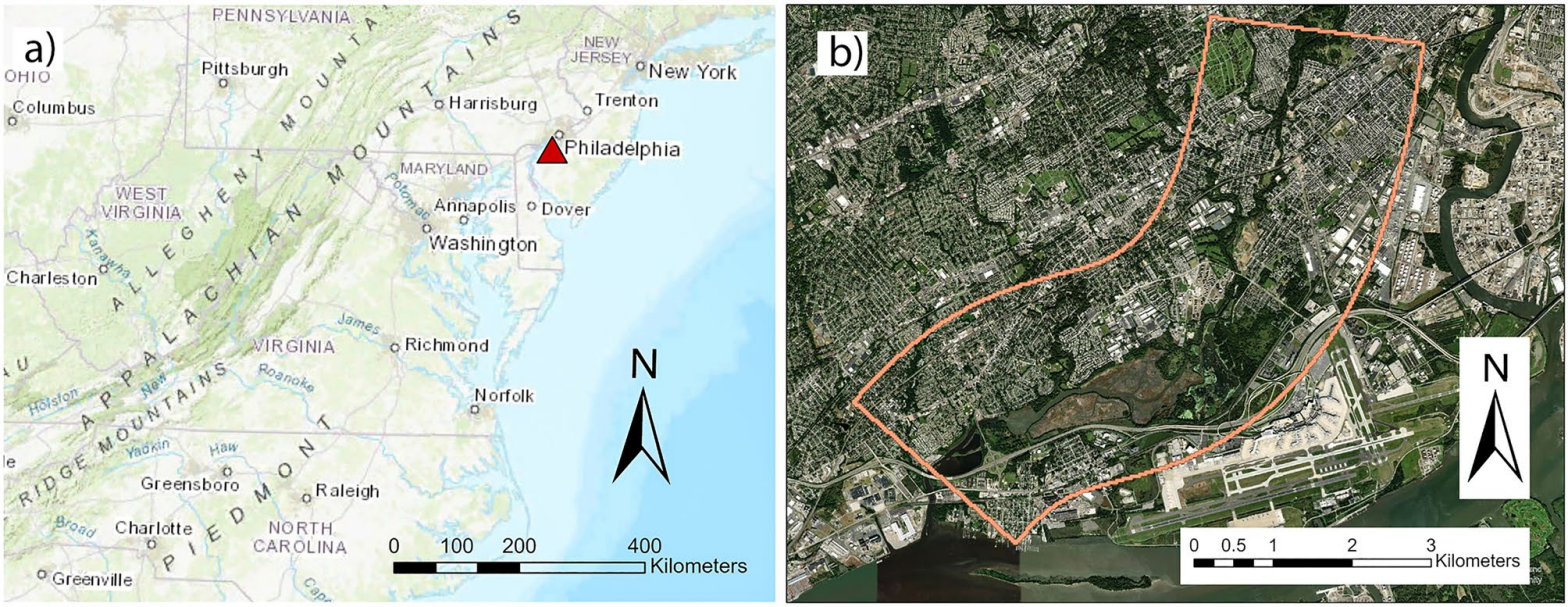
The figure above shows the global (**a**) and local (**b**) terrain of the study area. Maps are processed and generated in the ArcGIS Pro platform^81^.

The study area considered in this study is the lower part of Darby Creek (DC), along the southwest border of Philadelphia, PA, USA, shown in Fig. 1^77^. The alluvial channel of the Creek flows through a floodplain with fully urbanized settings which is subject to frequent flooding. The population residing near the creek is subject to flooding significantly^78^. The portion of the river considered in this study flows from the Mt. Moriah Cemetery (upstream) to the confluence with the Delaware River (downstream) and carries alluvial deposits through an urbanized setting^79^, approximately 15 river kilometers (rkm). Darby Creek plays an important role in the adjacent environment and ecology; it is also a flood-prone area^48^. It also offers a unique environment for various plant and animal species^80^.

### Preparing hydraulic dataset in iRIC

Hydraulic models are simulated in the iRIC platform to generate dataset for ML classifiers and DNN regression model. The iRIC is a numerical tool capable of modelling rainfall runoff generation, flooding, and sediment dynamics. It receives terrain and hydraulic data (e.g., water surface elevation, roughness) for the model calibration purpose. FaSTMECH (Flow and Sediment Transport with Morphological Evolution of Channel) is used as a solver in this study to model flood extent and depth^82^. Notably, this was a fluvial hydraulics model, and did not include a rainfall runoff simulation. Instead, model calibration used elevation data from the floodplain and bathymetry of the channel with water surface elevation at the upstream of USGS Cobb Creek gage at Mt. Moriah Cemetery (USGS gage 01475548) for the flooding event of 30th August of 2009 is utilized to calibrate the hydraulic model^77^. The terrain data is discretized to a size of 5 m^2^ for every computational cell. As the higher discharges from the upstream side of the river are responsible for the morphological changes, higher discharge values from the highest flood event in Darby Creek are chosen to create scenarios for AI models. Multiple scenarios are created using various constant discharge values upstream of DC within a certain range. The discharge data from observed flood events in the time span of 14th July to 16th September is obtained from USGS peak stream flow data (USGS gage 01475548)^83^. A set of discharge values is chosen to execute ML/DL models used in this study. The discharge values are 37, 42, 45, 50, 52, 61, 83, 95, 99 and 164 m^3^ per second (cms). The outcomes generated by the iRIC are water surface elevation and flooding depth. A set of urban hydraulic features i.e., the quantity of the impervious areas within the contributing area, and downstream distance from the hydraulic structures e.g., stormwater outfall and dam are introduced in this study to integrate the effect of urban attributes with the flooding extent and magnitude. Furthermore, the average slope of the contributing area is derived through GIS analysis and incorporated to represent the flow accumulation to a specific location.

### AI models

**Figure 2:**
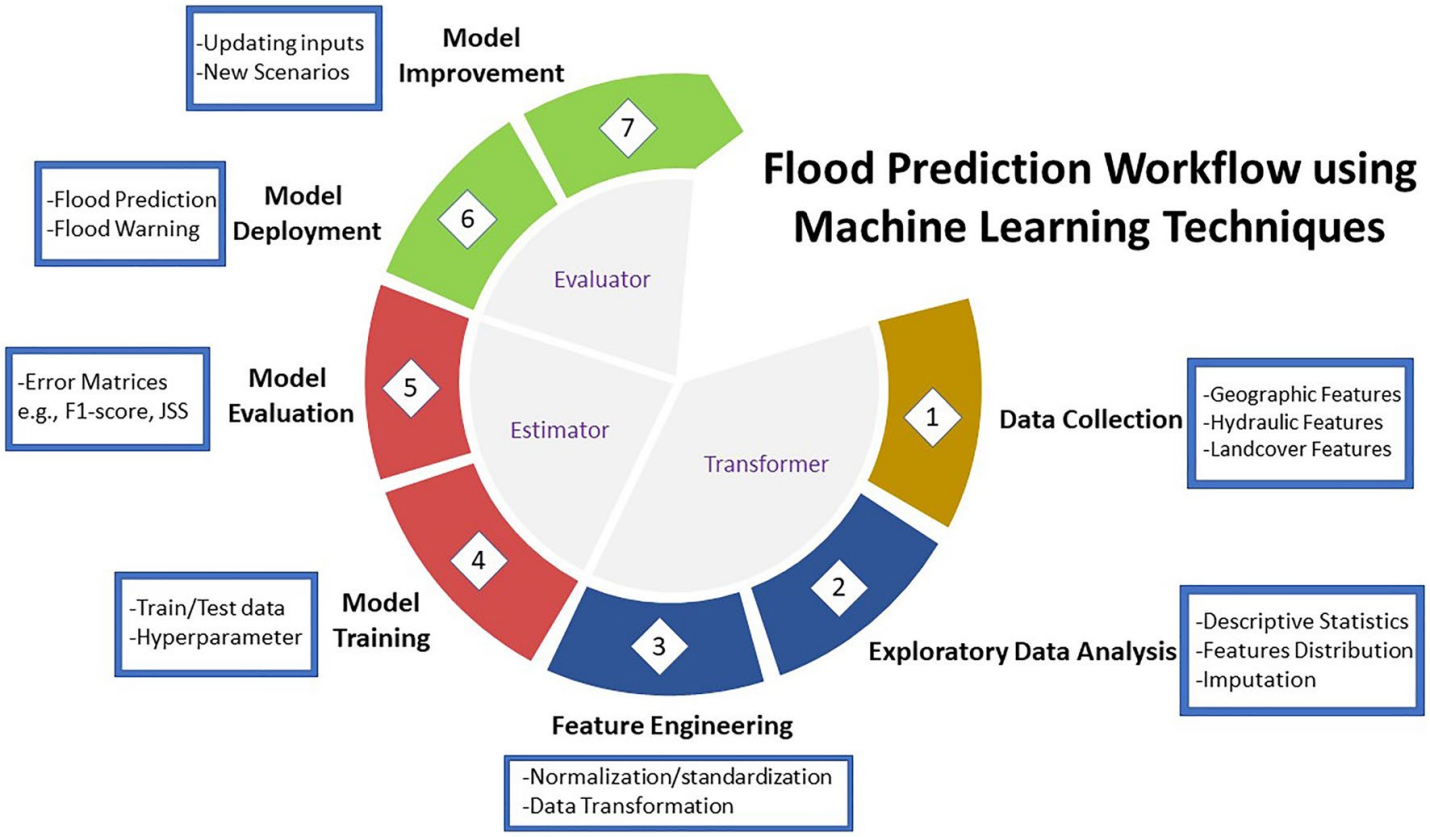
Flood Prediction workflow using machine learning classifiers and neural network regression techniques illustrates how the tasks are linked from the data preprocessing steps to the model deployment and maintenance stage.

The quantification of the flood extent and depth by the ML framework is accomplished in three steps. Firstly, exploratory analysis and feature engineering are performed to study and transform the entire dataset prepared by multiple geographic and hydraulic features that impact the hydrograph, listed in Table 1. After analyzing the dataset and conducting necessary transformation on the features, classifiers, such as Logistic regression (LR), K-nearest neighbors (KNN), decision trees (DT), support vector machines (SVM), are trained using the data prepared in the first step to locate or classify the flooded locations for each scenario of various upstream discharges. Third, a DNN is used to prepare a regression model to predict the depth of water within the computational domain. ML classifiers and DNN models are evaluated using several error matrices, e.g., F1-score, Jaccard similarity score and Root Mean Square Error (RMSE). The algorithms are tuned and optimized by altering the hyperparameters to reduce the error and obtain satisfactory performance. The ML workflow of flood prediction is described in Fig. 2. The entire process can be divided into groups of tasks, i.e., data collection, exploratory data analysis, feature engineering, model training, model evaluation, model deployment, and model improvement. Details are provided in the following sections. The steps are further categorized into distinct groups namely transformer, estimator, and evaluator.

**Table 1:** Full descriptions of the predictors and target variables used to train/test the ML classifiers and DNN regression model.

Features	Full descriptions
*x*_*1*_	x-coordinates of every location in the model domain
*x*_*2*_	y-coordinates of the same location
*x*_*3*_	Elevation in meters of same location
*x*_*4*_*/y*_*2*_	Depth of water in meters
*x*_*5*_*/y*_*1*_	Flooded locations
*x*_*6*_	Average slope of the contributing area of every point in percentage
*x*_*7*_	Number of impervious locations of the contributing area
*x*_*8*_	Downstream distance from the stormwater outfalls
*x*_*9*_	Downstream distance from the dams
*x*_*10*_	Upstream river discharge in m^3^/s

### Feature engineering

Within the first group of activities (transformer step), data collection, preprocessing, Exploratory Data Analysis (EDA) and feature engineering are performed (Fig. 2). EDA involves observing descriptive statistics and initial investigation of the variables (Table 1). Scikit-learn is used as the ML library for feature engineering in Python^84^. It offers several classifications, regression and clustering algorithms including LR, KNN, DT and SVM which are used as binary classifiers for identifying flooding locations in this study. Modules needed for ML and Deep Learning algorithms such as optimization, linear algebra, integration, interpolation, and special functions can be accessed through SciPy^41^. Independent variables for Binary Classifiers and DNN regression model are listed in the Table 1. Flooded location is used as the target variable denoted by y_1_ in case binary classifiers and water depth, y_2_ as a target variable in case of DNN model. Spatial information, coordinates and elevation values are obtained from the original Digital Elevation Model (DEM) of the study area using ArcGIS Pro. Water Depth and Discharge values are extracted through simulating multiple hydraulic models in the iRIC platform. Average Slope and number of impervious cells of the contributing area of every point of the DEM are urban hydraulic features, which have not been introduced before as a training feature for AI models. ArcPy, a Python site package that offers an effective and efficient way to perform geographic data analysis, data conversion, data management, and map automation using Python was utilized to generate the contributing areas of every cell upstream in the model domain^85^. It can be compared with the upstream area contributing to those cells. No modification was needed to alter the data type, as it is generated from iRIC-FaSTMECH simply as binary data type. The main data frame is constructed through concatenating datasets derived from different upstream discharges (Q) scenarios.

Feature Engineering tasks used in this study to prepare the datasets for the ML/DL algorithms include numerical imputation, outlier detection with standard deviation and dropping, splitting training/testing datasets, and scaling with normalization. The proportion of the train-test split is assumed to be 80/20 for both ML classifiers and DNN regression. Dataset is divided into train/test split in a non-reshuffle manner where the datapoints are selected for training purpose without random sampling from original dataset to make the test dataset independent from the train dataset. In Eq. 1 how the normalization of the features performed can be observed. *X* denotes the feature vector including all the features used to train/test the models. Preparation of dataset for training the DNN is identical to the preparation of the training dataset for ML Classifiers. Eighty percent (80%) of the data is used to train, and the rest of the data is used to test both the ML classifiers and DNN regression model.

1 $$X_{norm} = \frac{{X - X_{min} }}{{X_{max} - X_{min} }}$$

### Identifying flooded locations with ML classifiers

#### Logistic regression (LR)

In the Activity 4 in Fig. 2, ML classifiers and DNN model are trained using the independent variables (Table 1) to predict the flooded locations and depth. Linear regression searches a function that builds relationships to a continuous dependent feature/variable, **y**, to some outcome/predictors (independent features *x*_*1*_*, x*_*2*_*,* etc.). LR is a variation of linear regression, utilized when the existing dependent variable/outcome, ***y***_***1***_*,* is categorical. LG uses log loss as the loss/objective function in the classification algorithm. It generates a formula that forecasts the probability of the category as a function of the independent features. Logistic regression fits a special s-shaped curve (sigmoid function) by taking the linear regression and converting the numeric into a probability with the function, which is known as the sigmoid function σ^86^.

2 $$h_{\theta } \left( x \right) = \sigma \left( {\theta^{T} X} \right) = \frac{{e^{{\left( {\theta_{0} + \theta_{1} x_{1} + \theta_{2} x_{2} + ...} \right)}} }}{{1 + e^{{\left( {\theta_{0} + \theta_{1} x_{1} + \theta_{2} x_{2} + ...} \right)}} }}$$

The probability of a category 1 (a location being flooded) = (*Y* = 1|*X*) = $\sigma \left( {\theta^{T} X} \right) = \frac{{e^{{\left( {\theta^{T} X} \right)}} }}{{1 + e^{{\left( {\theta^{T} X} \right)}} }}$. Therefore, LR passes the features (e.g., *x*_*1*_ = elevation, *x*_*2*_ = slope of the contributing area, *x*_*3*_ = water depth, etc.)) through the logistic/sigmoid functions; however, considers the outcome as a probability. The goal of LR algorithm is to identify the best parameters *θ*, for ℎ(*x*) = σ(θ^*T*^*X*), in such a way that the algorithm forecasts a cell is being flooded or not in the model domain.

#### Decision tree (DT)

Decision tree learning is one of the predictive modelling approaches used in statistics, data mining and machine learning. It uses a decision tree (as a predictive model) to go from observations about an item e.g., features mentioned in the Table 1 (represented in the branches) to conclusions about the item's target value, e.g., binary decision on a location being flooded or not (represented in the leaves)^87^. From Scikit-Learn, Decision Tree Classifier is used to perform the classification task on flooding location. Gini Impurity is used as a loss function of the DT classifier^44^.

#### Support vector machine (SVM)

SVM works by mapping data to a high-dimensional feature space so that data points can be categorized, even when the data are not otherwise linearly separable. A separator between the categories is found, then the data is transformed in such a way that the separator could be drawn as a hyperplane. Following this, characteristics of new data can be used to predict the group to which a new record should belong. Like the LG classifier, SVM uses the logistic loss function with a piecewise linearization^88^.

#### K-Nearest Neighbors (KNN)

The principle of KNN is based on the concept that the k closest objects or similar cases in the p-dimensional space (the number of dimensions is identical to the number of the features mentioned in the Table 1) determine the class of an unknown variable i.e., flooded locations. KNN aims to partition n observations (number of rows in the flood prediction data frame) into k clusters tagging each observation (rows in the data frame) to a specific cluster with the cluster centers or cluster centroid or the nearest mean serving as a prototype of the cluster. The entire data space is partitioned into Voronoi cells in this approach. As the target variable (flood locations) is predicted by local interpolation of the target associated with the nearest neighbors in the training dataset with the independent variables, no specific loss function is used in the KNN classifier^46^. When features are obtained in different physical units with vastly varying scale, normalizing the training features and outcomes can improve the accuracy of the KNN algorithm as it depends on distance of the data points for the classification^47^.

### Predicting flood depth with DNN model

After predicting the flooded locations, DNN regression model is used to predict the water depth (*y*_*2*_). To do this, a full set of multiple hydraulic variables/features mentioned in Table 1 and flooded locations from ML models are used to train/test the DNN model.. Open-source library TensorFlow is used in this study work to construct DNN model as it has an excellent particular focus on the inference and training of DNN^48^. Training a model with TensorFlow typically starts by defining the model architecture.

**Figure 3:**
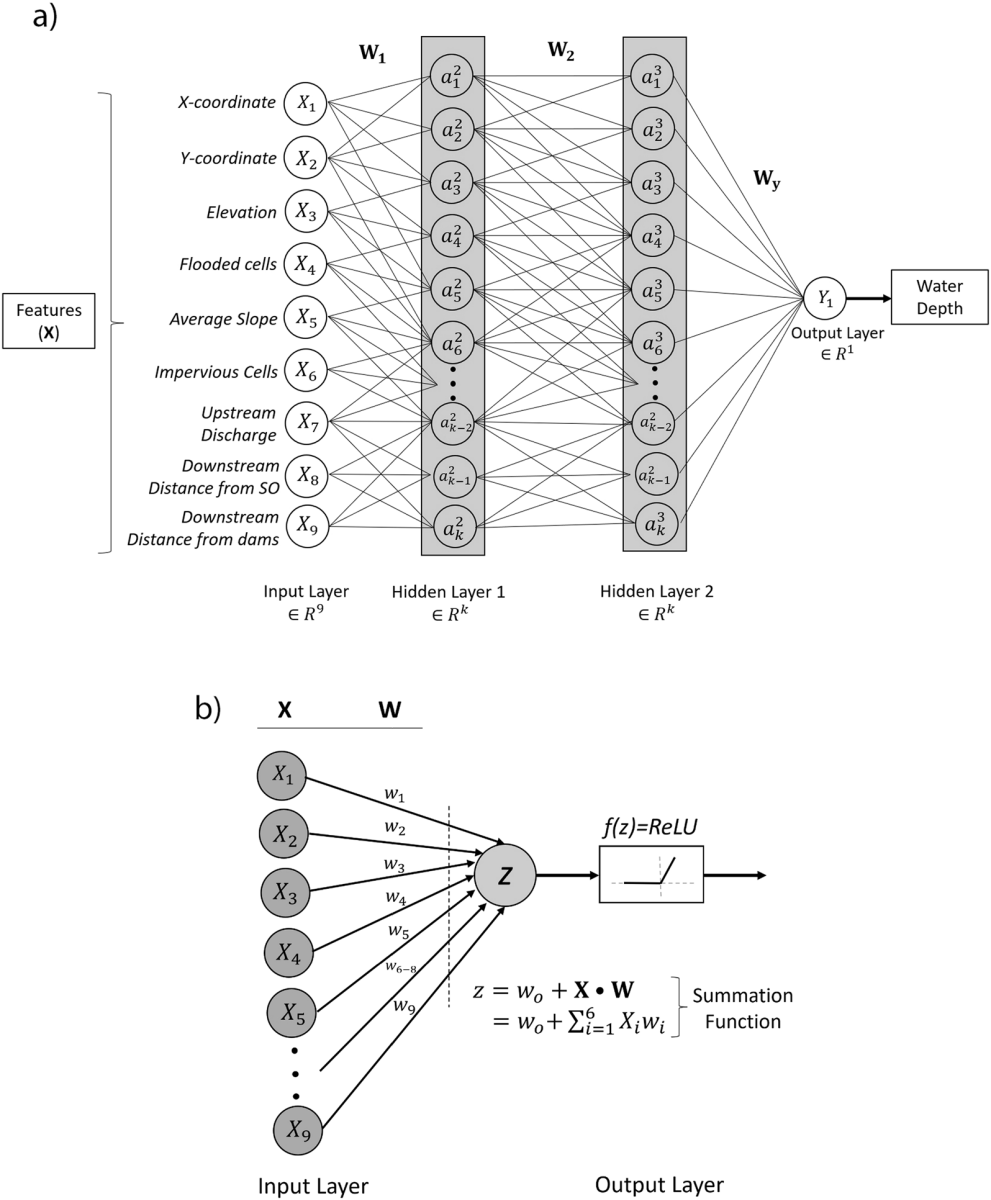
DNN architecture for flood prediction (**a**). A perceptron with summation of weights, features, and biases with the activation function (**b**). Input layers consist of all the input features and their corresponding weights. Aggregation of the features, weights and biases constitutes output layer.

Input layer contains features denoted by *x*_*i*_ in general, which is similar to the binary classification problem. The weights imposed on different features, aggregation of multiple features, further weights before the output layer and the activation functions are denoted with *W*, *z*, and a respectively. Finally, target variables (water depth) are generated from output layers. In Fig. 3 (a), introducing neural networks improves the prediction performance significantly through the introduction of non-linearity among the input and target features. The activation function used to introduce the non-linearity to the model is ReLU (rectified linear unit) function shown in Fig. 3 (b). This function returns the standard ReLU activation: maximum (*X*, 0), the element-wise maximum of input tensor (*X*) and 0 with default values. The total number of layers used to perform DNN is four including a normalized input feature layer, two hidden layers and a linear single-output layer. The total number of weights for each trainable neuron is 4609 where 11 neurons are found to be non-trainable.

**Figure 4:**
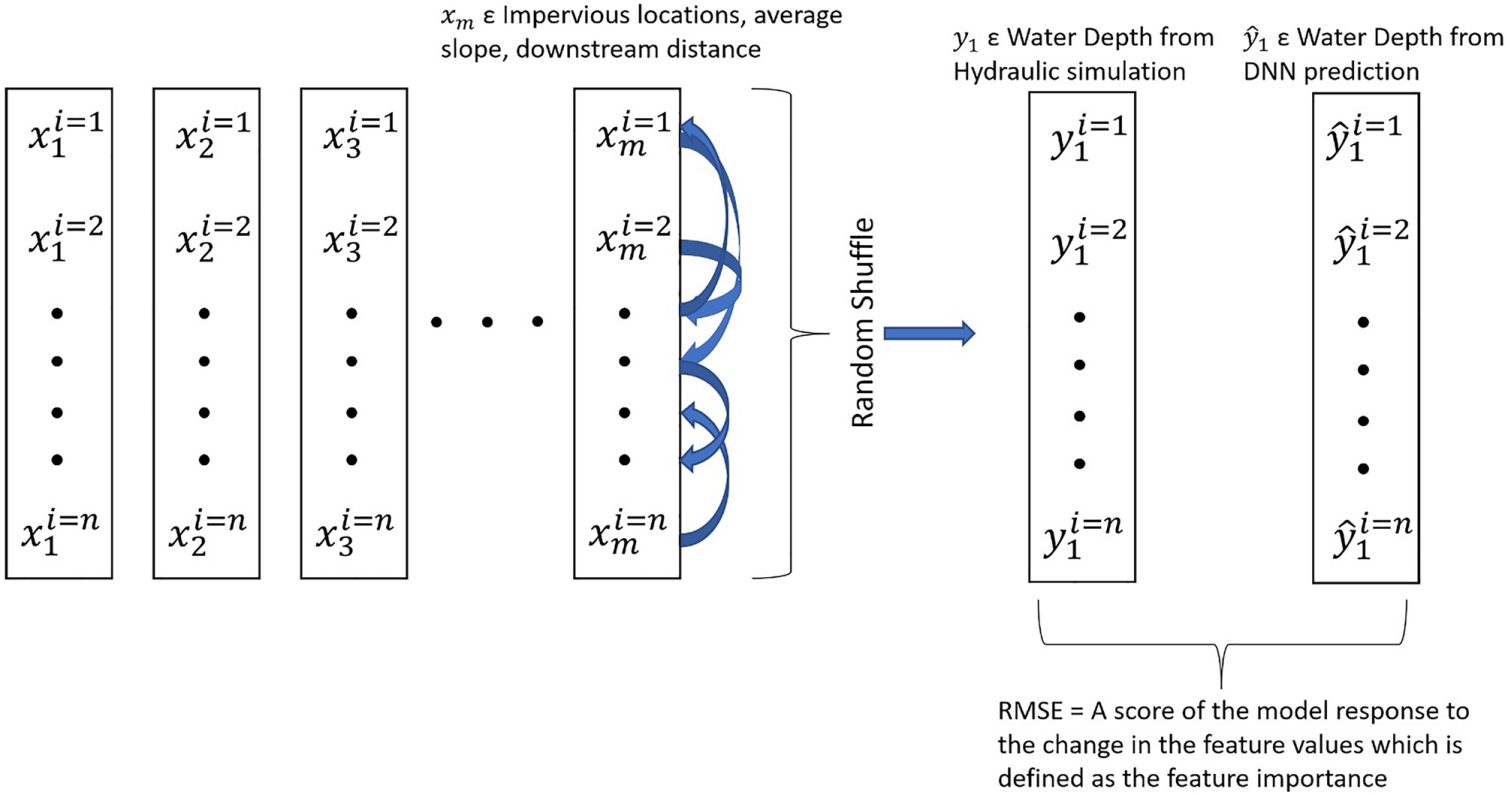
Mechanism of Permutation Feature Importance (PFI).

Urban hydraulic feature importance is studied by analyzing the sensitivity of the change in feature values over the target variable, water depth and Permutation Feature Importance (PFI) technique in the computational domain^89,90^ (Fig. 4). In PFI, the impact of shuffling the values of a feature, e.g., impervious locations (*x*_*m*_) within the contributing area over the target variable (${\widehat{y}}_{1}^{i}$) is quantified to observe the response in output variables due to the change in input variables. The score of the error matrix (RMSE) derived from the observed and predicted values of water depth as a result of the shuffle in the independent variable provides the score of feature importance. The values of impervious area, average slope of the contributing, downstream distance (DD) from the Stormwater Outfall (SO) and Dams (DO) are varied (5%, 10% and 20%) to observe the impact on the target variable in the DNN regression Model. The RMSE values are obtained from the difference between the series of the target variable, water depth after running the DNN model with the changed features and the series before running the model. In the PFI technique, DNN model is run with the values of a specific feature, e.g., impervious areas of the contributing area permuted/shuffled keeping the other features constant and the change in the RMSE values are recorded41. Only the output from DNN model i.e., water depth is used as a target variable in estimating the feature importance of other input variables as the output from ML classifiers are already used as an input variable in the DNN model.

### Model evaluation for the study area

In the activity 5, model evaluation in Fig. 2, ML classifiers and DNN model are evaluated for the entire study area using multiple error matrices. Several conventional statistical measures are available to evaluate the performance of the ML classifiers. Mean Absolute Error (MAE), F1-score, True Positive (TP), False Negative (FN), Root Mean Square Error (RMSE) Jaccard similarity score, log-loss are among the popular choices and provide evaluation of the models in quantitative terms^91^. In this study, F1-score and Jaccard similarity score are used to evaluate the ML classifiers. The F1-score is used to evaluate binary classification algorithms, e.g., logistic regression, which generates binary outputs of whether a location is flooded or not. The harmonic mean of the model’s precision and recall is calculated to determine the F1-score^92^. The performance of the ML classifiers can also be determined from the confusion matrix, shown in Fig. 8. A confusion matrix is a table that is used to define the performance of a classification algorithm. A confusion matrix visualizes and summarizes the performance of a classification algorithm. It is used to visualize the performance of a classifier, typically a supervised classification algorithm^93^. Two parameters needed to estimate the F1-score are precision and recall. Precision represents the fraction of the number of instances which the model correctly predicted (*T*_*p*_) and the sum of all instances that are incorrectly predicted as true (*F*_*p*_). Recall, sometimes referred to as sensitivity, is the fraction of the number of instances which the model correctly predicted (*T*_*p*_) and the sum of all instances that are incorrectly predicted as false (*F*_*n*_)^94^.

3 $$2*\frac{precision*recall}{{precision + recall}} = \frac{{T_{p} }}{{T_{p} + 0.5\left( {F_{p} + F_{n} } \right)}}$$

The Jaccard coefficient quantifies similarity between finite sample sets and is determined as the size of the intersection divided by the value of the union of the sample sets. Given forecasted values of fluvial flooding occurrence as ($\widehat{y}$) and actual values of flooding occurrence as y, the Jaccard index can be defined as

4 $$j\left( {y,\hat{y}} \right) = \frac{{y \cap \hat{y}}}{{y \cup \hat{y}}}$$

Root Mean Squared Error (RMSE) value is used for the DNN regression model evaluation. Datapoints are selected randomly from the entire study area to prepare the train/test sets. The train/test split used in this study is 80/20 providing 80% of the total datapoints for train set and 20% for test.

### Blocked K-folds cross-validation

**Figure 5:**
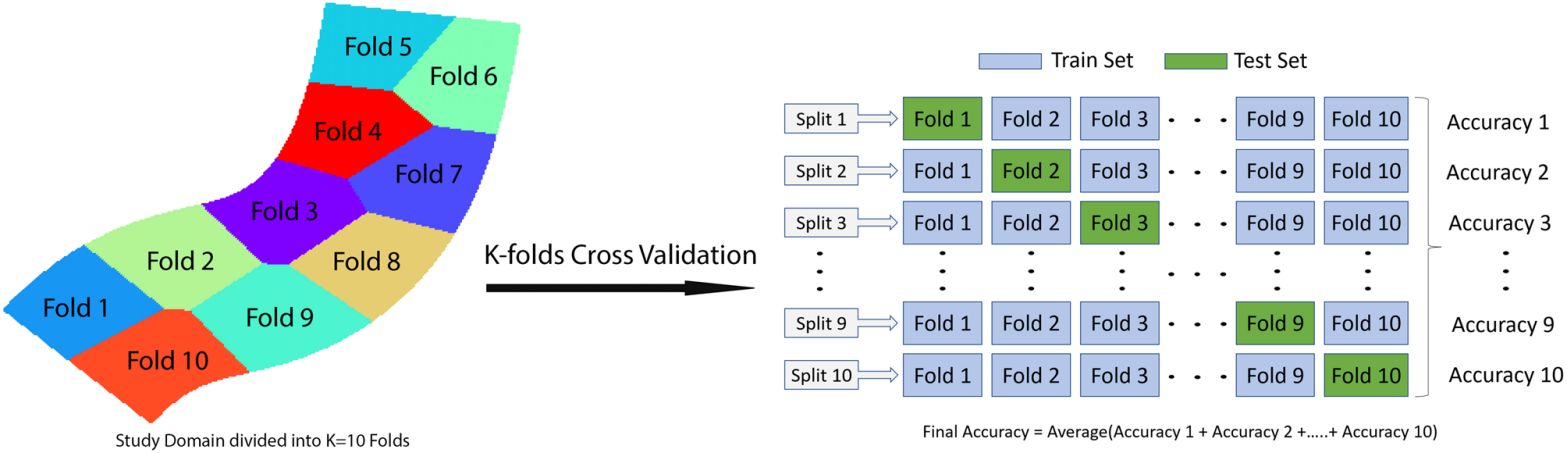
Blocked K-folds CV with a set of ten tarin/test splits.

Blocked (spatial) K-folds Cross-Validation (CV) is performed for the ML classifiers in classifying the flooded locations^95,96^. The entire study domain is clustered into 10 folds (zones) as spatial autocorrelation among the nearby cells may lead to bias and wrong model evaluation if the models are evaluated considering the entire study area only^97,98^. In the random sampling for train/test split, there is a possibility of taking the cells out in the study domain for the training set which are neighbors to the cells taken out for the test set. Consequently, those features in the train and test set are no longer independent, invalidating the evaluation of the ML classifiers. Therefore, the entire study domain is grouped into 10 folds to prepare individual train/test sets to compute the error matrix. Finally, the average of all the values of the error matrix is computed to show the model performance. An illustration of the entire process of the blocked K-folds CV is presented in Fig. 5. Ten different splits are introduced to isolate the test set with an independent fold. In each split the rest of the nine folds are used to train the model.

## Results and discussion

### Scenarios from hydraulic model

**Figure 6:**
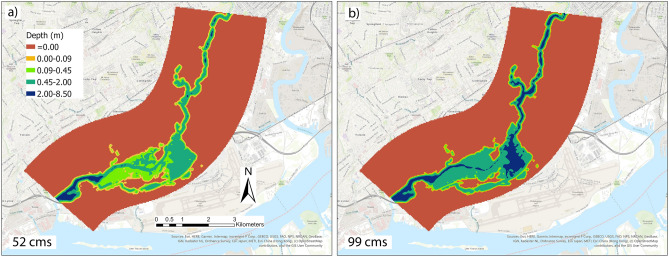
Flooding extent and depth estimated by the FaSTMECH solver in the iRIC platform for the scenarios with discharge values (**a**) 52 and (**b**) 99 cms. These scenarios are used to train/test ML classifiers and DNN regression algorithm. Maps are processed and generated in the ArcGIS Pro platform^81^.

The relationship with the water depth in the computational space and river discharge is highly non-linear. High variation in the geometry of the channel and roughness of both channel and floodplain against the flow can enhance the non-linearity in the system. In this section, water depth variations with their corresponding locations are presented with respect to multiple scenarios with several upstream discharge values, including 37, 42, 45, 50, 52, 61, 83, 95, 99 and 164 m^3^ per second (cms). The validated hydraulic model from iRIC is used to simulate and create scenarios having the water surface elevation and depth, locations, and binary output regarding a certain location is flooded or not as results. In Fig. 6, two plots of water depth with the locations for the scenarios with discharges 52 and 99 cms are shown, which are obtained from hydraulic simulation in iRIC.

### Binary flood map

**Figure 7:**
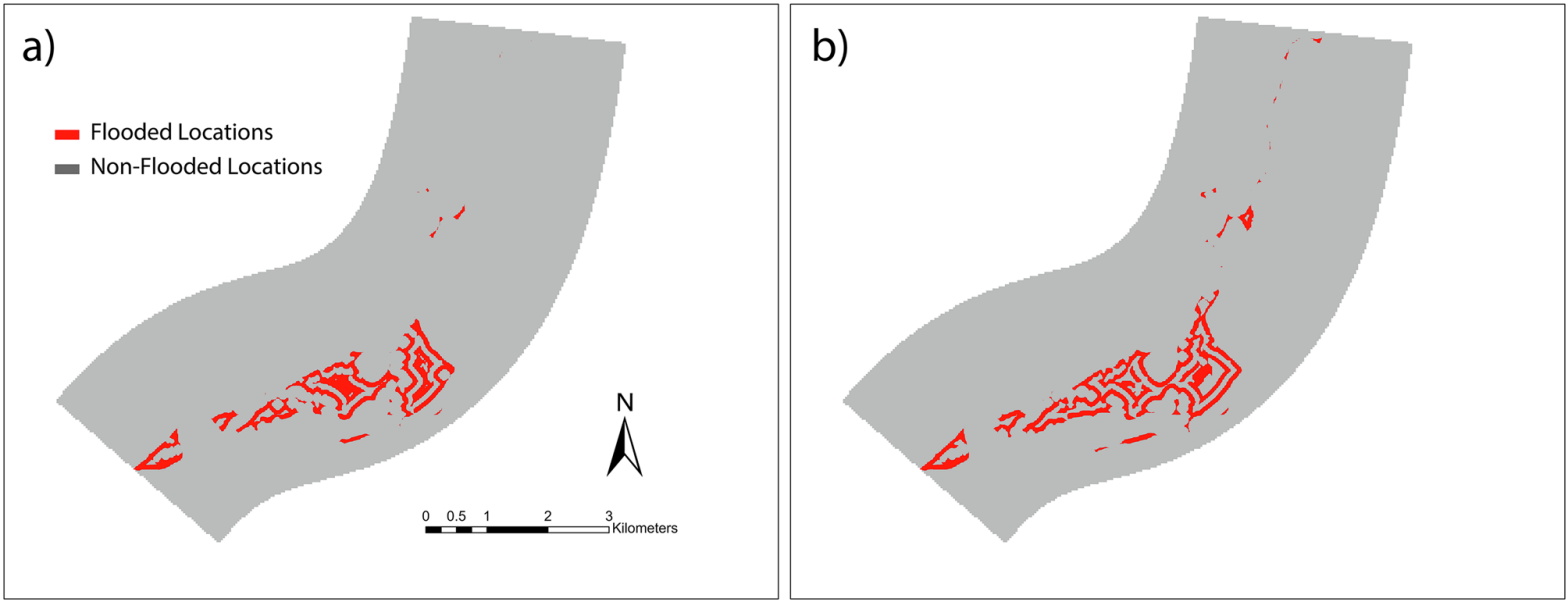
Flooded locations detected by the DT model for representative scenarios with upstream discharge values (**a**) 52 and (**b**) 99 cms. Prediction performance of the DT algorithm is the best among all other classifiers. Maps are processed and generated in the ArcGIS Pro platform^81^_._

The features used to train the ML classifiers (LR, DT, SVM and KNN) are *X* = [*x*_*1*_: x-coordinate, *x*_*2*_: y-coordinate, *x*_*3*_: ground elevation, *x*_*4*_: water depth, *x*_*6*_: average slope, *x*_*7*_: number of impervious locations, *x*_*8*_: downstream distance from SO, *x*_*9*_: downstream distance from dams, *x*_*10*_: upstream discharge]. Locations are classified using their corresponding water depth into two classes, whether a particular location is flooded or not (1/0). Figure 7 illustrates the distribution of flooded location for the scenarios with the upstream discharge values of 52 and 99 cms predicted with the DT algorithm. DT is the top performer among all other ML classifiers with the F1-socre and Jaccard Similarity matrix of 0.991 and 0.966.

All ML classifiers conveyed satisfactory performance in isolating flooded locations as the values of the error matrices in Table 2 are closer to unity. From Table 2, it can be observed that LR and DT outperform other classifiers. The F1-score and Jaccard similarity matrix of the LR and DT are 0.975, 0.991 and 0.995, 0.986 respectively which are greater than the values of the SVM and KNN.

**Table 2:** Comparison of the performances of ML Classifiers.

Binary classifiers	F1-score	Jaccard similarity score
Logistic Regression (LR)	0.975	0.995
Decision Tree (DT)	0.991	0.966
Support Vector Machine (SVM)	0.892	0.901
K-Nearest Neighbors (KNN)	0.855	0.810

**Figure 8:**
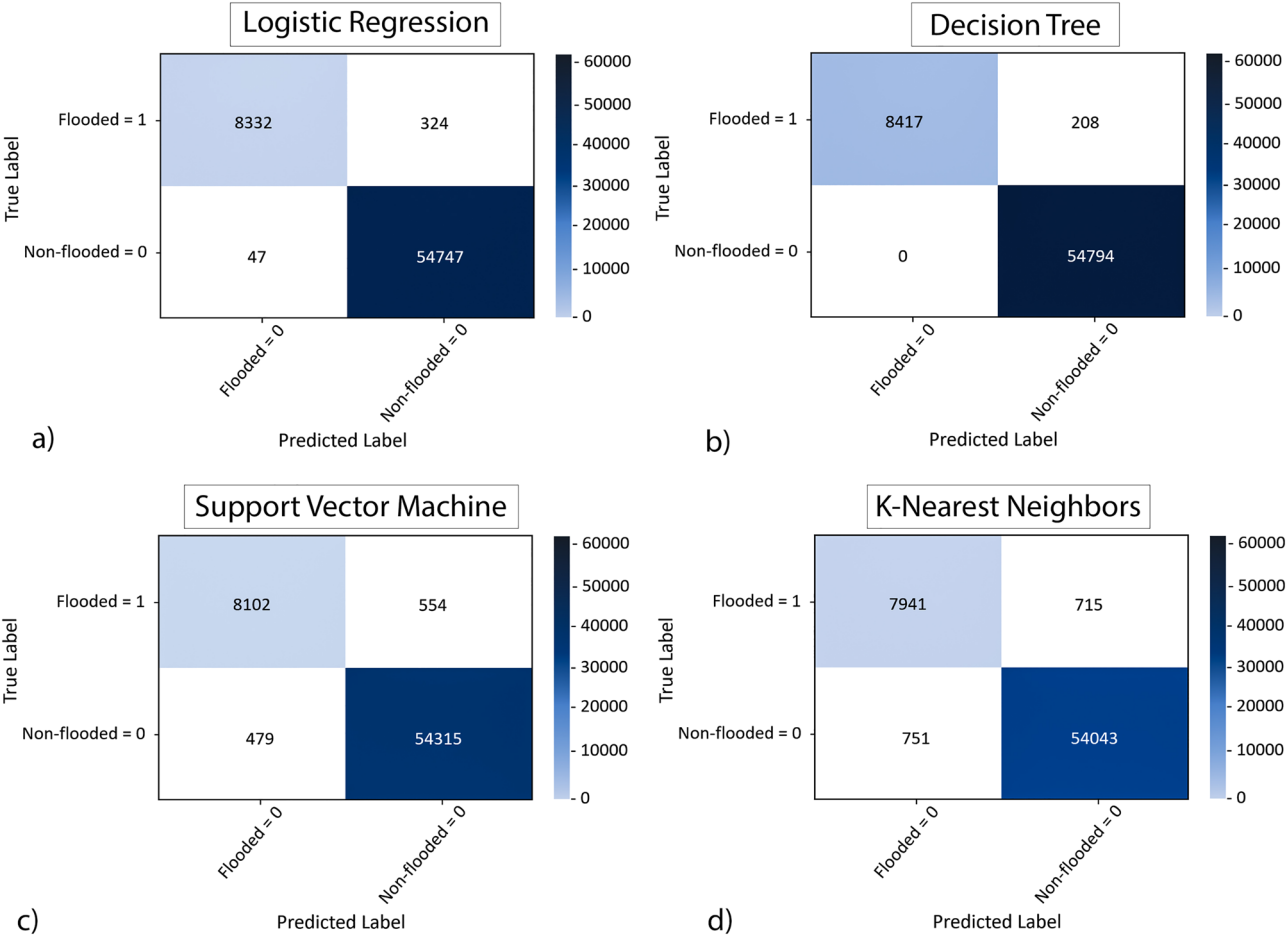
Confusion Matrix is used to observe the performance of ML classifiers i.e., logistic regression (**a**), decision tree (**b**), support vector machine (**c**) and K-nearest neighbors (**d**). Total number of predicted cells from ML classifiers are compared to the actual values from hydraulic model to extract the number of correctly predicted cells.

Performance of the binary classifiers can also be illustrated in the form of confusion matrix, comparing correctly predicted outcomes with the incorrectly predicted outcomes. In Fig. 8, the confusion matrices showed to illustrate the performance of the ML classifiers. The number of flooded cells predicted correctly by LR algorithm (a) numbers 8332 (96.8%), while the incorrectly predicted cell count is 324 which is significantly lower than the number of correctly predicted locations. Similarly, the number of correctly predicted not-flooded cells count 54,747 (99%) where the number of incorrectly predicted not-flooded cells are 47. A total of 96.8% of predicted cells are correct, suggesting a highly accurate model performance.

**Figure 9:**
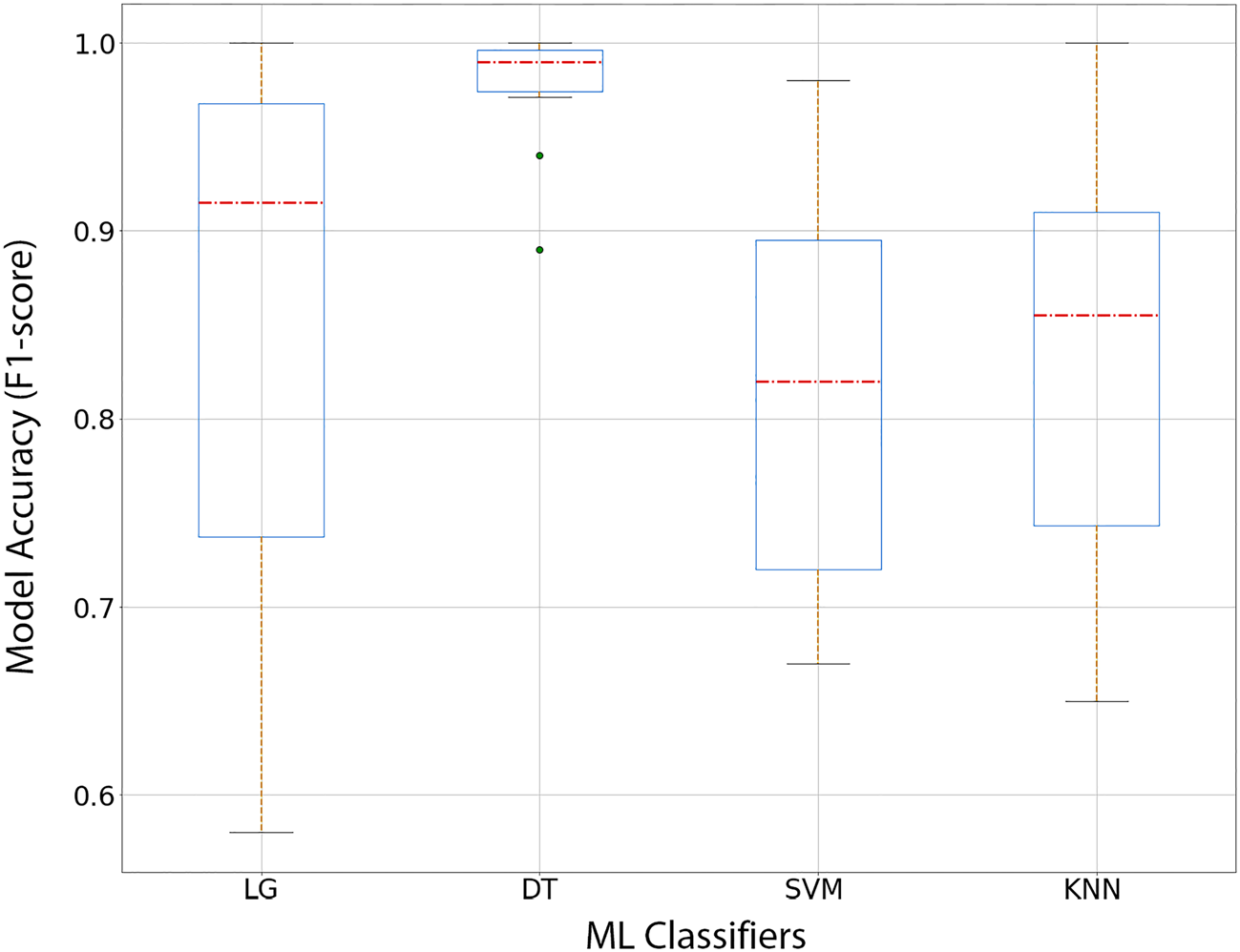
Distribution of the model accuracy (F1-score) in K-folds CV for the ML classifiers.

The distribution of the individual model accuracy of the ML classifiers in the blocked K-folds CV is presented in Fig. 9 using boxplots. Median values of the model accuracies (F1-score) are presented with the red lines with the values 0.924 (LR), 0.975 (DT), 0.827 (SVM) and 0.871 (KNN). The range of the accuracy scores of the DT algorithm is 0.890–1.000 and is found to be the best ML classifier among all others. The median score of the DT model is 0.975 and is the highest score followed by the LG, KNN and SVM. Overall, LG, DT, KNN, and SVM showed satisfactory performance, as the range of the model accuracy of all classifiers is 0.582–1.000. As the dataset used for the predictive analysis is a tabular dataset with segmented values of the input variables, as well as a categorical-typed target variable (flooded/non-flooded) in this case, tree-based non-parametric algorithm i.e., DT algorithms, outperform other classifiers by capturing the interaction between different features. However, extensive hyperparameter tuning, intermediate feature creation and variation in the size of dataset might lead to alteration in model performance. However, extensive hyperparameter tuning, intermediate feature creation and variation in the size of dataset might lead to alteration in model performance.

### DNN regression to predict flood depth

Artificial neural network with a single hidden layer is not capable of extracting the insights of the non-linearity and complexity of the flood prediction. Therefore, DNN with multiple hidden layers is incorporated in the prediction process of water depth. Adding more hidden layers increases the accuracy of prediction. However, inclusion of a large number of hidden layers requires high computational power and may result in overfitting the model^99,100^. From the model evaluation, it can be observed that the DNN described in this manuscript reflected the complexities of river flood prediction. To capture the high amount of non-linearity among the geographic and urban hydraulic features mentioned in this paper and establish linkage among them, it is a prerequisite to introduce multiple hidden layers. Hidden layers with the nodes built in them are used to train the model through an iterative optimization process. A total of three hidden layers are used with 64 neurons assigned to each. While the number of epochs found best with a minimum error is 110. 80 percent of the whole dataset was used for model training purposes, while 20 percent was used for testing the performance of the DNN. The activation function for hidden layers used is ReLU. Other popular activation functions such as hyperbolic tangent, sigmoidal or leaky ReLU functions are recommended to introduce the non-linearity in DNN. The model evaluation matrix, RMSE value of 0.027 illustrates the DNN regression algorithm conveyed satisfactory performance in resolving the high non-linearity in the flooding depth prediction process.

**Figure 10:**
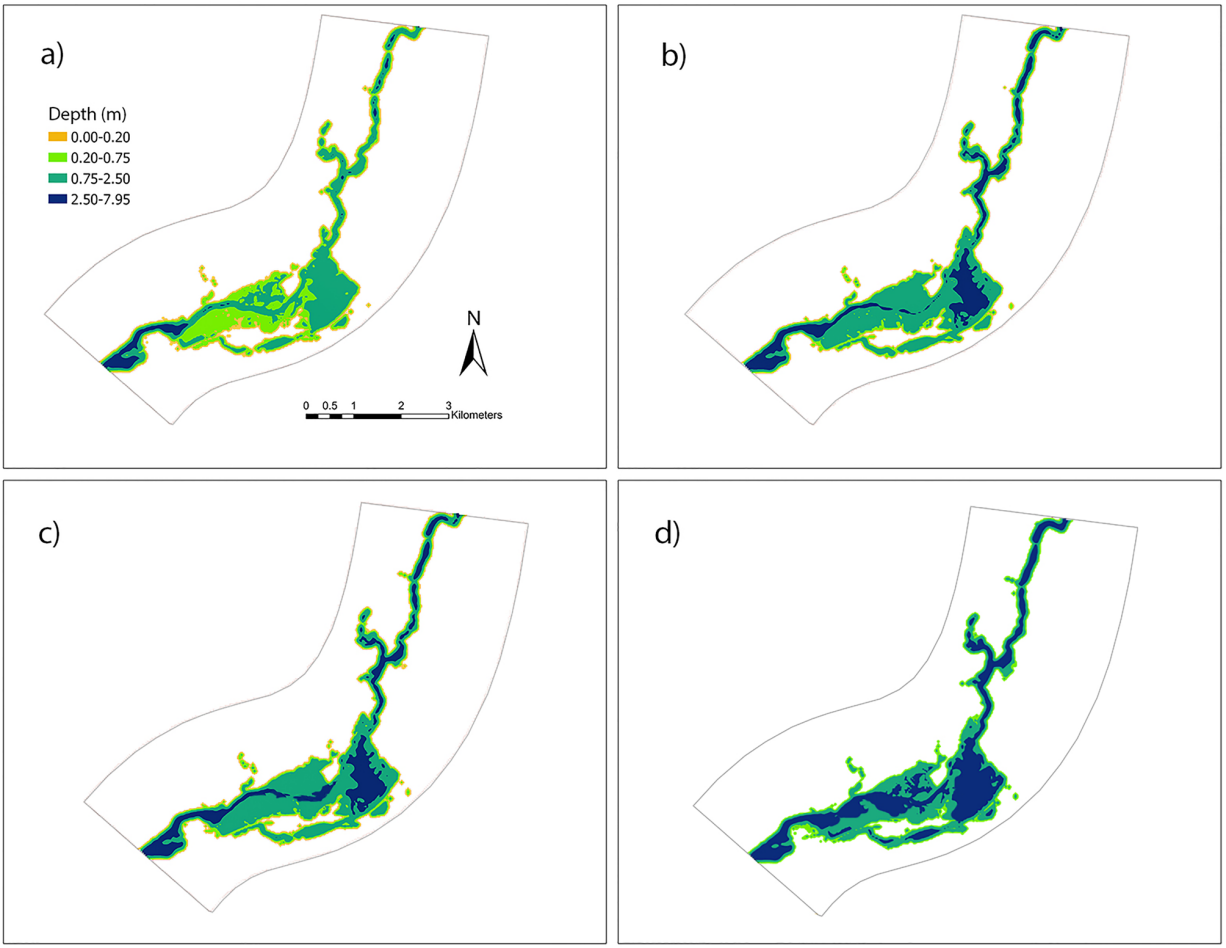
Predicted water depths for the representative scenarios covering the full range of upstream discharge values (**a**) 42, (**b**) 52, (**c**) 99 and (**d**) 164 cms outputs from the DNN regression model. Maps are processed and generated in the ArcGIS Pro platform^81^_._

Urban hydraulic features, i.e., average slope and the number of impervious cells of the contributing area, are introduced in this process to train the DNN regression model. It is clear from Fig. 10 that the flood depth is highly correlated and sensitive to the upstream discharge. With the increase in the upstream discharge, flood depth also increases. In the DNN regression model training phase, a non-linear correlation is built among these urban hydraulic features and flooding amount and extent. By introducing these urban hydraulic features, connections among the features and target variables i.e., number of impervious locations and water depth are established.

**Figure 11:**
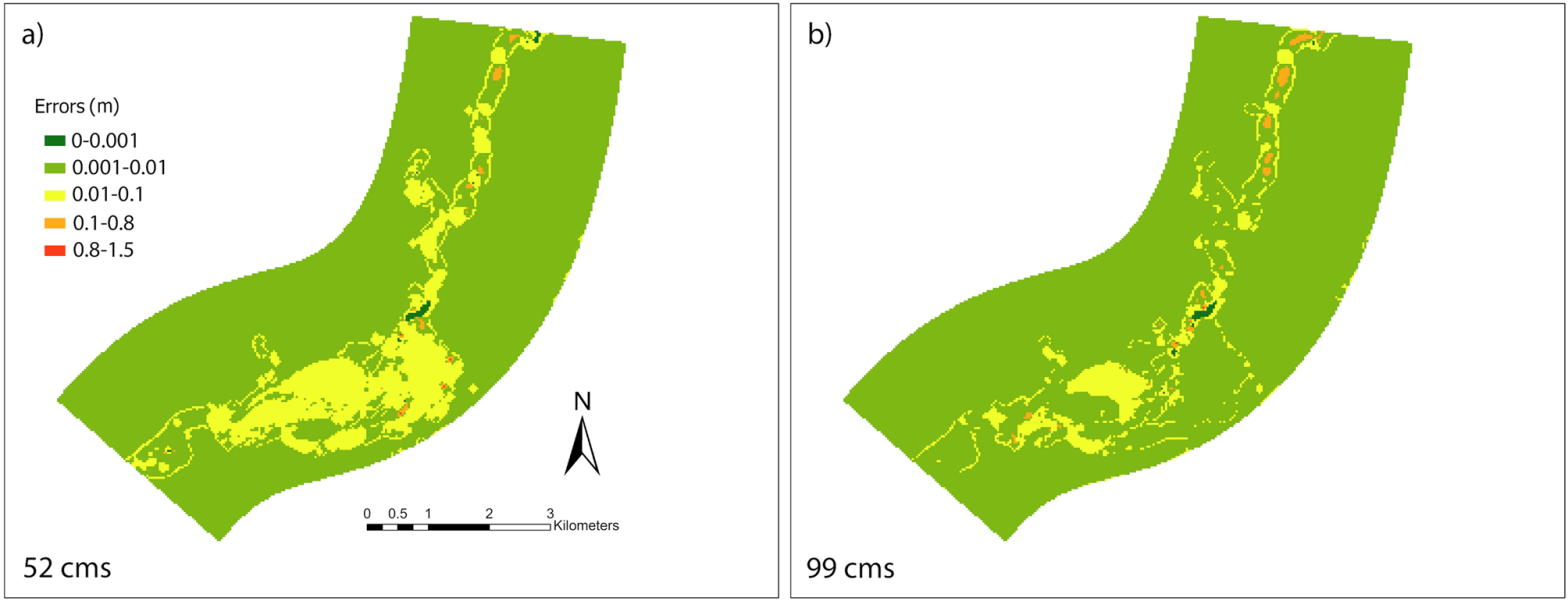
Error distributions calculated by subtracting the water depth from DNN regression model output and iRIC simulation is shown here for two scenarios with upstream discharge of 52 (**a**) and 99 (**b**) cms. Maps are processed and generated in the ArcGIS Pro platform^81^.

As the output (flooded location) from the ML classifiers is used in the DNN model, CV is not performed for the DNN model evaluation. However, difference mapping (error distribution) of the water depth simulated/predicted in the hydraulic and DNN model is presented in Fig. 11 in addition to the model evaluation for the entire study domain with the error matrix, RMSE. The difference maps in Fig. 11 illustrate the variation in the predicted water depth from DNN regression model and hydraulic model. The spatial distribution of the differences is not significant (in the scale of 0.01 m) for varying upstream discharge condition. Based on the error matrices and difference mapping, it can be concluded that the performance of the DNN regression model is excellent for the urban hydraulic features considered in this study. Orange locations showing high range of difference in errors (predicted and observed flood depth from iRIC) were found mostly in deep water region along the stream obtained from the hydraulic simulation. They are also surrounding the downstream potion of the stream with greater transverse extent with deeper water level. DNN model conveyed a few wider ranges of error in predicting comparatively deeper water along the stream. Imposing more weights on the high water level (extreme values) to reduce the error might lead to a better prediction performance by DNN in the deep-water zones.

**Figure 12:**
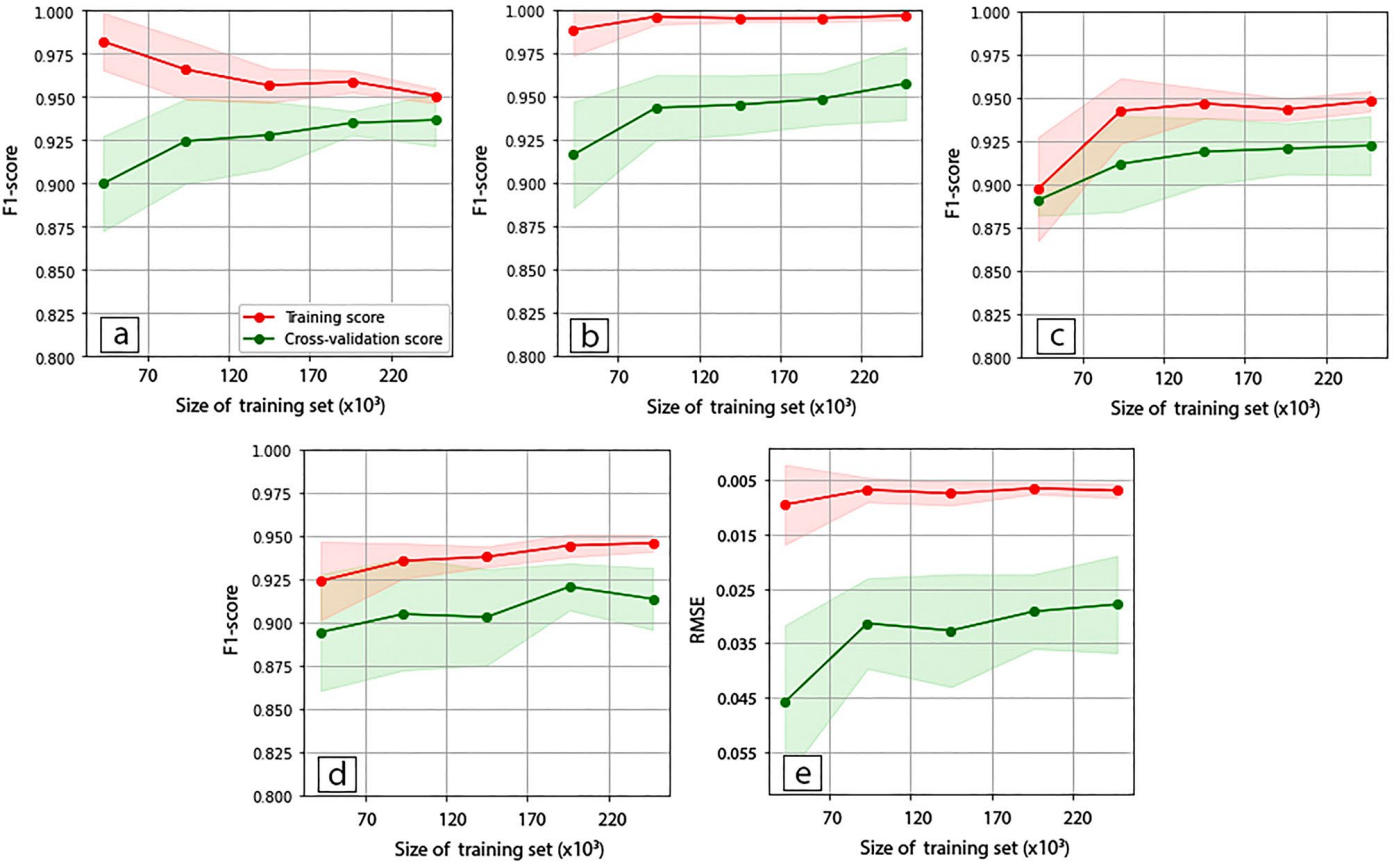
Performance learning curves of the ML classifiers, (**a**) LG, (**b**) DT, (**c**) SVM, (**d**) KNN and (**e**) DNN model with the training (red) and cross-validation score (green).

Performance learning curve (PLC) of the ML models conveys the impact of the size of the training examples over the model performance. In the Fig. 12, PLC is shown with the train and cross-validation scores for the ML classifiers in flood detection and DNN model in quantifying flood magnitude. Among the ML classifiers, DT performed best with a top F1-score of 0.994 in training and 0.959 in cross-validation. Model performance improve for all models with the increase in the training phase except LR shown in the Fig. 12 (a). Poor performance of the LR model may result from the overfitting, bias, algorithm incompetence in capturing the complexity in data and overall data quality issues. Future studies could focus on the point of diminishing returns for the size of training data in the context of physics-guided AI models in flood detection. Similar to the ML classifiers, DNN model shows improved performance (lower RMSE) with the increase in size of the trainset. The total number of datapoints (examples) to train the models is 253,800 which is 80% of the total datapoints of 317,250 with 8 m of spatial resolution in the computational domain. In case of DNN model, the performance score, RMSE of 0.027. Training sample size is dependent on the spatial resolution of the computational domain. Increasing the resolution may lead to increase in the size of the dataset (increasing the size of the trainset). However, that may end up with less computationally efficient estimation.

### Urban hydraulic feature importance

**Figure 13:**
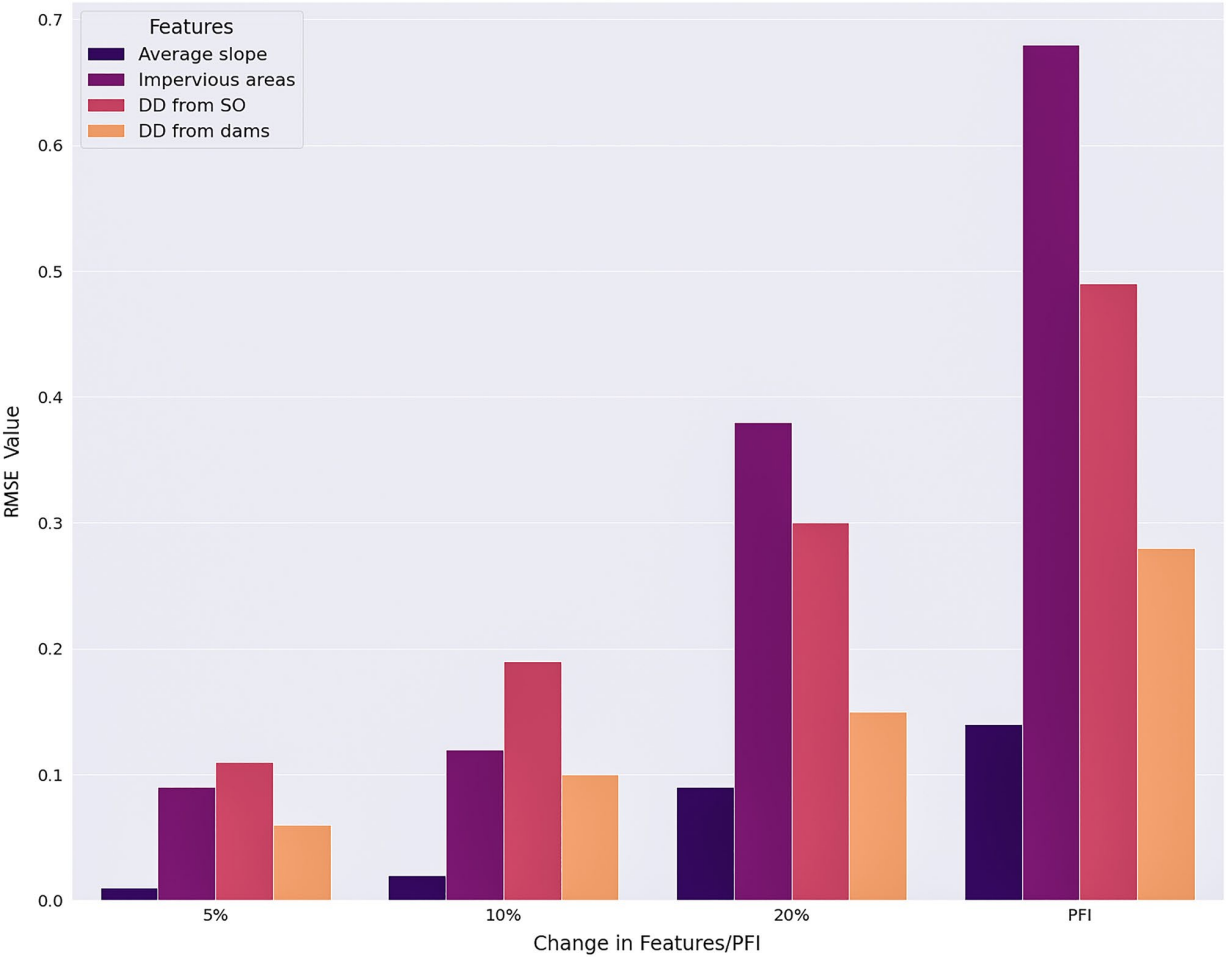
Change in the RMSE value of the DNN regression model due to the % increase/decrease in the feature values and Permutation Feature Importance.

Permutation Feature Importance (PFI) and sensitivity analysis approach are used to determine the impact of the urban features on the DNN regression prediction output i.e., water depth based on the RMSE value as the indicator. PFI measures the variation in the prediction error of the model after the feature’s values are permuted^71^. This approach quantifies the change in the RMSE values as the prediction error after a series of feature values of interest is permuted/shuffled breaking the linkage between the feature (e.g., downstream distance from stormwater outfall) and target variable (e.g., water depth). This measure is an indicative of the dependency of the model outcome to a specific feature. A sensitivity analysis for the increase in the feature values (5%, 10% and 20%) is performed to quantify the response of the target variable in the DNN regression model, i.e., water depth to the variation in the urban features using RMSE value. From both analysis, impervious area is found to have the highest importance to predicting flood depth compared to the other features. This is logical, as impervious areas directly contribute to runoff and hence to the accumulation of water. The importance scores in both approaches are shown in Fig. 13 to illustrate the significant response of water depth predicted from the DNN regression model due to the change in the urban hydraulic features. The iRIC model used was a hydraulic fluvial model, and did not include rainfall runoff, but the model was calibrated with USGS gage data and validated with NDWI data, showing a high spatial accuracy for flood inundation^77^. Impervious areas showed the highest influence over the target variable i.e., the water depth predicted from the DNN regression model followed by the downstream distance of the stormwater outfall and dams and average slope to the contributing area in case of PFI and 20% increase in the feature values. For the 5% and 10% increase/decrease in the feature values, downstream distance from the stormwater outfall showed highest impact. The score of the feature importance, the RMSE value increases for all features with the increase in the change of the feature values from 5 to 20% shown in Fig. 13. Highest response in the RMSE value can be observed in the case of PFI where the feature values are permuted instead of adjusted with a simple sensitivity analysis by percent increase. While these values show the relative importance of features contributing to flood inundation spatially, the disconnection with the physics may create a scenario where the driver(s) of fluvial flooding is limited.

## Conclusion

Robust and real-time prediction of flooding is critical to alleviating the growing risk of urban fluvial flooding. Estimation of the water depth of the river and floodplain for various scenarios is of paramount importance in urban flooding planning and management, particularly as many municipalities seek to install or upgrade infrastructure. Data-driven ML approaches provide a path to circumvent the complexities of urban flooding using geographic and urban features outlined in this paper and have the potential to get insights into the flooding attributes. Computationally expensive physics-based numerical models become burdensome at the city-scale and beyond. Traditional hydraulic models depend on solving physics-based differential equations, they require extraordinarily processing power and high memory allocation, specifically for large amount of data and thus perform much slower compared to data-driven methods presented in this paper. In this study, flooding in urban areas, such as the highly urban Darby Creek watershed, is predicted using hybrid physics-informed data-driven techniques. A novel approach to classify and predict the flooded locations and depth using various ML classifiers and DNN-based regression method illustrates a promising ground and potential to entirely shift into the data-driven techniques. Derived urban hydraulic features, i.e., impervious locations and average slope within the contributing area, downstream distance from the stormwater outfall and dams, are introduced in this paper to incorporate the unique impact of urban features on the riverine.flooding extent and magnitude which was not present in the previous research works. Future inclusion of additional parameters and resolutions can aid in deepening the understanding of urban hydrology.

A set of binary classifiers (LR, DT, SVM, KNN) is used to identify the flooded locations and a DNN regression model with multiple hidden layers is applied to capture the high non-linearity and quantify the flood magnitude in an urban environment. Both the classification and regression algorithms trained to predict the flooding locations and depth in urban areas with minimum error generated satisfactory outcomes. All error matrices used to evaluate the performance of the binary classifiers are F1-score, Jaccard Similarity matrix and confusion matrix delineate the promising capability of the ML classifiers in isolating flooded locations. The RMSE value used to evaluate the adequacy of the DNN algorithm in predicting water depth. also showed satisfactory performance for the unique datasets with geographic, derived urban and physics-informed hydraulic features. Further, the urban hydraulic feature importance scheme quantified the impact of urban features over the outcome (water depth) of the DNN regression model. Therefore, the satisfactory performance of the proposed framework presented here shows a higher potential for flood prediction in an urban environment, by accounting for the influence of urban features compared to the traditional physics-based hydraulic models. In addition, due to spatial autocorrelation effect, random sampling of data points to prepare the train/test splits from the entire study domain may not yield a satisfactory validation of the model to generalize. In this study, a blocked K-folds CV is performed to further validate the model's performance to generalize to the unseen areas. The performance score for CV showed satisfactory performance for all the spatially segmented blocks as well as the entire study area. Proposed CV framework can be useful in validating ML based flood models to generalize the model performance. For improved flood estimation in complex urban area, a balanced perceptive of the proposed framework could serve as a discerning tool for the engineers and decision makers. Data itself cannot be an alternative for physical modeling, however, when combined with the informed and detailed knowledge of the physics-transformed variables from hydrodynamics models, it is highly likely to yield more precise and comprehensive solutions.

The computational time required to converge to the solution by the physic-based model iRIC has been reported approximately one and half times higher (1 h 7 min for iRIC; 32 min for ML models) than the full pipeline of training ML models for flood detection, estimation and feature importance with the ML classifiers and DNN regression model. However, average time required by the trained ML classifiers was 4.7 s in estimating the flood extent where it was 9.3 s for the DNN model in quantifying the flood magnitude. Trained AI models can also be highly efficient in reproducing a range of scenarios which may aid to the decision-making process in a faster and more efficacious way compared to the hydrodynamic model. In addition, several influencing factors (e.g., average slope and impervious locations of the contributing area, downstream distance from the stormwater outfall and dams) used in the ML classifiers and DNN model, were not taken into consideration in the physic-based model equations to quantify the flood extent and magnitude. Therefore, in both, the computational time required and inclusion of the number of influencing factors to the target variable, ML models outperformed the physics based iRIC simulation. The performance of the ML classifiers and DNN regression models can be improved with the increase in the discretization of the computational domain creating more training and testing data. Geographic and hydraulic features can be stored in the web where the entire training or testing workflow is possible to be executed in the cloud-computing platform. Further, other machine learning and deep learning classifiers and regression models such as Gaussian Process classification, Bayesian classification, Histogram-based gradient boosting, and Long Short-Term Memory regression can be studied with the river hydraulic dataset. Notably the transferability of this method is data-limited. Linking to physical models has the potential to advance model capabilities, as well as allow for deeper insight into urban hydrologic processes, Future work in this area is highly recommended as the data availability and computational power are increasing rapidly. The approach outlined in this study has the potential to be combined with the weather forecast models paving the way of feasible and inexpensive quantification of real-time riverine flooding scenarios.

## Supplementary Information

Supplementary Information.

## Data Availability

Data collected for the study can be made available upon request from the corresponding author.
